# Glutamatergic Signaling Along The Microbiota-Gut-Brain Axis

**DOI:** 10.3390/ijms20061482

**Published:** 2019-03-25

**Authors:** Andreina Baj, Elisabetta Moro, Michela Bistoletti, Viviana Orlandi, Francesca Crema, Cristina Giaroni

**Affiliations:** 1 Department of Medicine and Surgery, University of Insubria, via H Dunant 5, 21100 Varese, Italy; andreina.baj@uninsubria.it (A.B.); m.bistoletti1@uninsubria.it (M.B.); 2 Department of Internal Medicine and Therapeutics, Section of Pharmacology, University of Pavia, via Ferrata 9, 27100 Pavia, Italy; elisabetta.moro@unipv.it (E.M.); francesca.crema@unipv.it (F.C.); 3 Department of Biotechnology and Life Sciences, University of Insubria, via H Dunant 5, 21100 Varese, Italy; viviana.orlandi@unisubria.it

**Keywords:** microbiota-gut-brain axis, glutamate, glutamate receptors, dysbiosis, irritable bowel syndrome (IBS), inflammatory bowel disease (IBD)

## Abstract

A complex bidirectional communication system exists between the gastrointestinal tract and the brain. Initially termed the “gut-brain axis” it is now renamed the “microbiota-gut-brain axis” considering the pivotal role of gut microbiota in maintaining local and systemic homeostasis. Different cellular and molecular pathways act along this axis and strong attention is paid to neuroactive molecules (neurotransmitters, i.e., noradrenaline, dopamine, serotonin, gamma aminobutyric acid and glutamate and metabolites, i.e., tryptophan metabolites), sustaining a possible interkingdom communication system between eukaryota and prokaryota. This review provides a description of the most up-to-date evidence on glutamate as a neurotransmitter/neuromodulator in this bidirectional communication axis. Modulation of glutamatergic receptor activity along the microbiota-gut-brain axis may influence gut (i.e., taste, visceral sensitivity and motility) and brain functions (stress response, mood and behavior) and alterations of glutamatergic transmission may participate to the pathogenesis of local and brain disorders. In this latter context, we will focus on two major gut disorders, such as irritable bowel syndrome and inflammatory bowel disease, both characterized by psychiatric co-morbidity. Research in this area opens the possibility to target glutamatergic neurotransmission, either pharmacologically or by the use of probiotics producing neuroactive molecules, as a therapeutic approach for the treatment of gastrointestinal and related psychiatric disorders.

## 1. Introduction

A complex bidirectional route of communication exists between the gastrointestinal tract and the brain, termed the gut-brain axis [1]. Hippocrates, the Father of modern Medicine, already in 400 B.C., has been quoted saying that “death sits in the bowel” suggesting that alterations of the gut functions may have detrimental consequences on the human body, including the brain. However, scientific straightforward evidence demonstrating the influence of the gastrointestinal tract on human health have been provided only from the nineteenth century. The gut-brain axis ensures proper coordination and maintenance of the digestive tract to support different ergotropic physiological functions but may also have profound effects on the central nervous system (CNS) development and on different aspects of behavior relevant to normal and pathological states. Conversely, it is now evident that the CNS, via this bidirectional axis, may control several gastrointestinal functions in normal and disease states [1,2]. Neuronal, hormonal and immune signaling affect the communication between the gut and the brain. The intestinal saprophytic flora participates to the modulation of these signaling pathways and is considered an effective component of the gut-brain axis, now defined as the microbiota-gut-brain axis [2,3]. The gut microbiota influences the maintenance of the body health homeostasis, both locally and systemically. In the gut, the saprophytic commensal flora controls several metabolic functions, the development of the immune system and the defense against pathogenic microorganisms, however, its effects extend from the gut to the CNS, since it is fundamental for brain development and homeostasis [3,4,5]. Alterations in the symbiotic relationship between the microbiota and the enteric microenvironment may thus have multiple consequences, including development of gut, behavioral and cognitive disorders [3,5,6]. In this perspective, the possibility to clarify neurobiological mechanisms along the microbiota-gut-brain axis, underlying control of host homeostasis, is fundamental and different molecular pathways are now explored. It is now evident that several neuroactive molecules, such as gamma aminobutyric acid (GABA), serotonin (5-HT), dopamine, noradrenaline and glutamate (Glu) are generated by both the eukaryotes and prokaryotes, giving rise to an interkingdom communication system. The present review will provide a description of the more recent evidence suggesting that glutamatergic pathways may participate in the modulation of the interkingdom communication, in physiological and disease conditions. In the gut, Glu, principally deriving from dietary proteins and from free Glu contained in food additives is a multifunctional amino acid involved in taste perception, intermediary metabolism and energy production [7]. In addition, a fraction of the free Glu in the lumen originates from bacterial synthesis [8]. Glu plays also a fundamental role as an excitatory neurotransmitter in the CNS and in the periphery, including the enteric nervous system (ENS), where the amino acid is synthesized by neurons and glial cells [9,10]. Increasing evidence suggests that modulation of glutamatergic receptors along the microbiota-gut-brain axis may influence multiple physiologic responses both in the brain and in the gut and alterations of the glutamatergic transmission may bear important consequences in the development of pathologies involving derangement of this communication axis [8,10]. Interestingly, metabolomic studies evidenced that changes in the gut saprophytic microflora are correlated with alterations in Glu brain levels [11,12]. Dysfunction in the microbiota-gut-brain axis has been correlated, for example, with the development of major gut disorders, irritable bowel syndrome (IBS) and inflammatory bowel disease (IBD), which are characterized by elevated psychiatric co-morbidity [13,14]. Research in this field opens an exciting scenario on the possibility to target the glutamatergic neurotransmission, by means of traditional pharmacological approaches as well as by the use of neuroactive molecule-producing probiotics, as new potential therapeutic tools, addressed to the treatment of neurogastrointestinal and/or psychiatric disorders. In this review, we will focus on the possible involvement of changes in glutamatergic signaling along the microbiota-gut-brain axis in the development of both IBD and IBS. 

## 2. Gut Microbiota and Host Interaction

The gastrointestinal tract harbors about 3.8 × 10^13^ bacterial cells belonging to approximately 2000 species, and other microorganisms, such as, virus, archaea, fungi and protozoa, representing the most abundant microbial population in the human body [15,16]. The gut microbiota represents an essential organ for the host homeostasis and as such has become the subject of many investigations carried out by multidisciplinary approaches in the field of nutrition, gastroenterology, endocrinology, immunology, neuropsychiatry and neurology [3,4]. Infants acquire their gut microbiota during intrauterine life: Firmicutes, Tenericutes, Proteobacteria, Bacteroidetes and Fusobacteria phyla were identified in placenta microbiota niche [17]. Furthermore, human colostrum provides a continuous supply of commensal and potential probiotic bacteria to the infant gut: staphylococci, lactic acid bacteria (LAB) and bifidobacteria, have been isolated from human milk samples. Interestingly, the same bacterial strains have been found in both breast milk and infant feces of different mother-infant pairs, confirming the role of human milk on the bacterial colonization of the infant gut [18]. However, the composition of gut microbiota changes throughout life depending on many factors: dietary, age, gender, genetics, geography, health, pharmacological treatment, hygienic conditions, lifestyle [19]. These factors influence the environment alongside the gut and select bacteria adapted to gastrointestinal niches depending on their optimal growth chemical and physical parameters (oxygen tension, pH, nutrient availability and concentration, water activity, temperature). The composition, diversity and abundance of human microbiota gut, in health and disease conditions, is under continuous evaluation by metagenomics approaches [20]. In healthy subjects, the most representative bacterial phyla are Firmicutes and Bacteroidetes; Proteobacteria, Actinobacteria and Verrucomicrobia are less abundant [21,22].

The symbiosis between the gut microbiota and host is the result of a dynamic equilibrium in which both get advantages. In this scenario, exponentially growing studies focus the attention on microbiota and human health and the intriguing interkingdom communication developed between prokaryotic and eukaryotic cells. Microorganisms harboring the human gastrointestinal tract produce many compounds useful to the host, such as vitamins, gas, organic acids, bile salts, bacteriocin [23,24]. The commensal saprophytic flora strengthens host innate and acquired immunity, representing a biological barrier against pathogens. On the other side, the gut represents the best environment, from a nutritional and physical viewpoint, underlying bacterial metabolic activities. Thus, the human gut is a “melting pot” where metabolites, cellular components, hormones, virulence factors, autoinducers, are released by prokaryotic and eukaryotic partners. The gut is one of the best examples of a human district where an elaborate interkingdom communication takes place.

The microbial contribution to this crosstalk has been greatly investigated. Although the host exploits bacterial metabolites for anabolic and catabolic functions, many compounds have other physiological effects. Amino acids released by bacteria can be used for host biosynthetic aim, and their deamination causes the accumulation of ammonia, carbon dioxide and short chain fatty acid (SCFA), influencing host physiological functions [25]. For example, SCFA, together with other metabolites, polyamine (i.e., putrescine, spermidine, spermine) and aryl hydrocarbon receptor ligands, influence the immunity response [26]. Many bacterial structural components such as lipopolysaccharide, lipoteichoic acid, peptidoglycan, flagellin, formyl peptides and unique nucleic acid structures mediate the cross-talk with the immune system [26]. Furthermore, the extended plethora of microbial virulence factors (i.e., pigments, proteases, nuclease, toxins, haemophores) represents a family of molecules detrimental to the host health [27]. It is now evident that the effect of bacterial “messengers”, released at the gut level, may extend from the gastrointestinal tract to more distant sites, including the brain [28].

## 3. The Microbiota-Gut-Brain Axis

**Figure 1:**
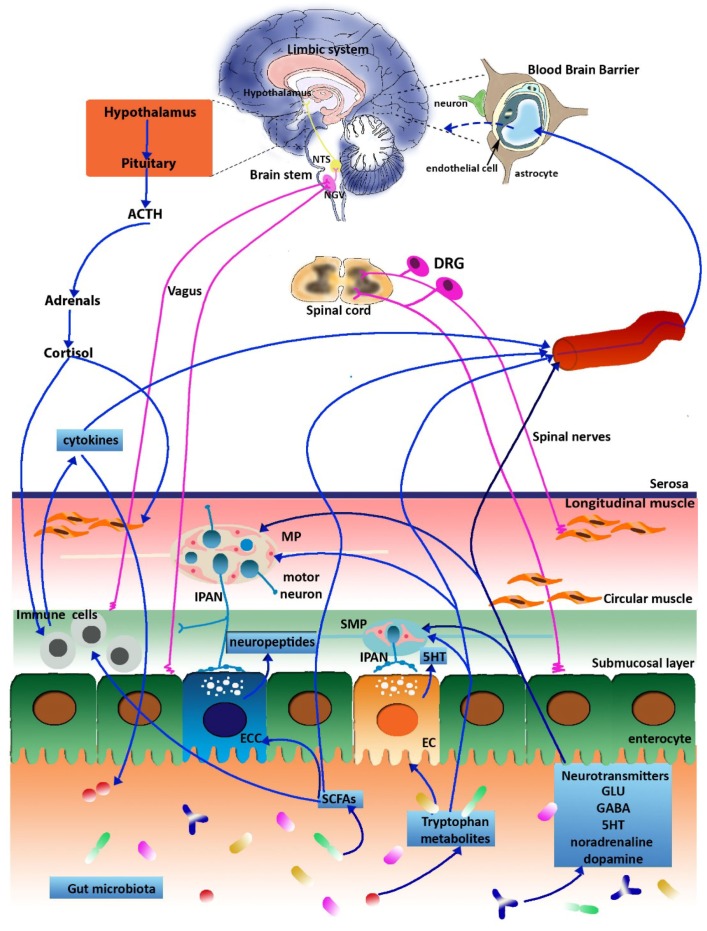
Schematic representation of the microbiota-gut-brain axis. The gut saprophytic microflora can signal to the central nervous system (CNS) and to the enteric nervous system (ENS) via different pathways, including endocrine, immune, metabolic and neuronal pathways explained throughout the text. With the exception of gamma aminobutyric acid (GABA), in normal conditions, the blood brain barrier impedes access of circulating neurotransmitters into the CNS, including Glu. However, when the blood brain barrier is disrupted, the levels of Glu, both in blood and brain markedly increase (dashed blue line). Abbreviations: NTS, nucleus of the solitary tract; NVG, nodose vagal ganglion; DRG, dorsal root ganglion; MP, myenteric plexus, IPAN, intrinsic primary afferent neurons, SMP, submucosal plexus, ECC, enteroendocrine cell; EC enterochromaffin cells, SCFA, short chain fatty acid (adapted from Mazzoli and Pessione, 2016 [8]).

A complex reflex neural network participates in the formation of the gut-brain axis, allowing a two-way communication system between the gut and the brain, which are in constant cross-talking both in health and disease [1,2] (Figure 1). Such bidirectional communication allows sensory visceral signals from the gut to influence the brain in order to regulate reflex activity and mood states, in turn, inputs from the brain may modulate several gut functions such as motility, secretion and the immune function [1,29]. The connecting neuronal pathways consist of afferent and efferent neurons, proceeding through the parasympathetic (vagal) and sympathetic (splanchnic and pelvic spinal pathways) branches of the autonomic nervous system (ANS). Afferent vagal neurons, whose cell bodies are contained within the nodose vagal ganglion (NVG), transmit sensory information to the nucleus of the solitary tract (NTS) in the brain stem regarding the presence of food, motor activity and degree of gut distension. This information is then sent to higher centers, e.g., hypothalamic areas, in particular to the paraventricular nucleus, which is the main source of corticotrophin releasing factor (CRF) or, more locally, to form long vago-vagal reflexes [29,30,31]. Efflux of efferent vagal signals via the dorsal motor nucleus of the vagus (DMV), underlays control of motor and secretory gut functions [29,30,31]. Afferent spinal neurons, whose cell body are contained in the dorsal root ganglia (DRG), participate in transmitting sensory inputs to the dorsal horn neurons of the thoracic and upper lumbar spinal cord, which then project to the CNS via spinothalamic pathways, and represent the main pain signaling pathways in the gut-brain axis [30,31]. In the CNS, both spinal and vagal afferent inputs synapse with higher brain regions, in particular with the emotional motor system, consisting in the limbic system and in some paralimbic structures (including the medial prefrontal cortex, amygdala, and hypothalamus), which coordinate responses to emotion along the gut-brain axis [30,31]. The hypothalamic–pituitary adrenal (HPA) axis, the main stress axis in mammals, participates to this bidirectional communication by releasing corticotrophin-releasing factor (CRF), which promotes the release of adenocorticotrophin hormone (ACTH) from the pituitary, which passes into systemic circulation to cause the release of cortisol in man from the adrenal glands. There are numerous reports suggesting that this hormonal cascade has an important role in the regulation of several functions of the brain–gut axis, particularly during stress, such as gastrointestinal transit, visceral sensation and permeability of the intestinal wall [1,30]. A peripheral component of this bidirectional communication axis is represented by the enteric nervous system (ENS), a complex neuronal network innervating the gastrointestinal tract, which receives sensory inputs from the ANS and transmits information to it [29]. Glu is recognized as neurotransmitter/neuromodulator involved in the regulation of several functions along the gut-brain axis, during both physiological conditions and disease states [10]. The amino acid, via activation of its receptors on either vagal, splanchnic or pelvic afferents, participates in conveying sensory inputs to brain areas involved in the regulation of different gut and brain functions, while efferent pathways drive both excitatory and inhibitory inputs into the gastrointestinal tract, may also be regulated by glutamate receptor activation [10,29,30].

In recent years, preclinical and clinical studies have highlighted the fundamental influence that the enteric microbiota exerts on the gut-brain axis, which is now renamed “microbiota-gut-brain axis” [2,28]. Several microbially-derived molecules may control different gut functions, including metabolic, nutritional and immune responses, but also brain activity, giving rise to a microbiota-mediated bottom-up control of the CNS [28]. For example, bacterial metabolites, such as SCFAs, stimulate enteroendocrine cells (EECs) of the gut epithelium to produce several neuropeptides, including peptide YY, neuropeptide Y, cholecystokinin, glucagon-like peptide-1 and -2 and substance P that, diffusing through the lamina propria, gain access to the bloodstream and/or local receptor, thus affecting intrinsic ENS neurons or extrinsic vagal innervation [32,33]. The saprophytic microflora may also regulate the production of neuroactive molecules, including classic neurotransmitters and neuromodulators. For example, brain levels of tryptophan (precursor of 5-HT), tyrosine (precursor of dopamine and noradrenaline) and glutamine (involved in the synthesis of both GABA and Glu) are lower in GF animals (GF, animals demonstrably free from microbes throughout their lifetime) [11,12]. Recently, the administration of probiotics to BALB/c mice induced a long-lasting enhancement of Glu/glutamine brain levels. Since, in physiological conditions, the blood brain barrier impedes the amino acid passage into the CNS, this data suggest that the gut microbiota may control defined biosynthetic enzymatic pathways involved in Glu production in the brain [8,28,34,35]. The gut microbiota may also indirectly influence glutamatergic pathways along the microbiota-gut-brain axis, by controlling L-tryptophan metabolism. In the gut, the essential amino acid L-tryptophan contributes to the synthesis of numerous bioactive molecules, including 5-HT, kynurenine (Kyn) and indole derivatives, under both direct and indirect microbiota control [36]. Tryptophan intake is conveyed into the Kyn pathway principally upon activation of the rate-limiting enzyme indoleamine-2,3-dioxygenase1 (IDO1) [36,37]. Two downstream by-products of Kyn are represented by KynA and quinolinic acid. Quinolinic acid is an agonist at the N-methyl-D-aspartate (NMDA) Glu receptor and has neurotoxic proinflammatory properties [38]. KynA is a natural antagonist at the glycine site associated with NMDA receptors [5,39,40] and is able to reduce excitotoxic damage, playing a neuroprotective role both in the CNS and in the ENS [39,41]. In this view, tryptophan metabolism along the Kyn pathway may have important implications in the control of the gut-brain axis signaling [37].

## 4. Sources of Glutamate along the Microbiota-Gut-Brain Axis

### 4.1. Dietary Sources of Glutamate

Dietary Glu represents a major source for Glu in the gastrointestinal tract and is the most abundant (8%–10%) among dietary amino acids [7]. The main sources for luminal Glu are diet protein and flavor-enhancing food additives, such as monosodium Glu (MSG). In addition, free dietary Glu may be naturally present in some food (aged cheeses, seafood, and some vegetables) [42]. In the lumen, the amino acid is internalized from the apical membrane of enterocytes via selective transporters, the most abundant being represented by excitatory amino acid carrier C1, EAAC1 [43]. In the large intestine, however, Glu is adsorbed from the arterial blood by colonocytes and the transfer of the amino acid from the colonic lumen to the portal circulation is almost negligible [44]. Both in humans and in different animal models, the 75%–96% of enteral Glu is removed during the first pass effect in the splanchnic bed, and above the 80% of this Glu contributes to the production of energy, necessary for supporting intestinal functions [45,46]. Glu may also participate to gut protein synthesis and, in the form of carbon plus nitrogen donor, to a number of fundamental metabolic pathways, including the synthesis of essential amino acids, such as proline and arginine, citrulline and the protective molecule, glutathione [47]. Studies carried out in preterm infants, adult humans, pigs and piglets indicate that such extensive luminal Glu metabolism limits the systemic absorption. Indeed, the amino acid reaches the systemic circulation in low concentrations (10–50 μM) compared to other amino acids, preventing the access of excessive amounts of ingested Glu into particularly sensitive districts, such as the brain [7]. Although, in normal conditions, dietary Glu does not enter the blood brain barrier [48], it is to be ascertained if changes in the barrier permeability induced by several factors, including stress, diet and gut microbiota alterations, may allow the transfer of the luminal amino acid into the CNS [8,49,50].

### 4.2. Bacterial Production of Glutamate

Glu is produced by several bacterial strains, many of them representing environmental bacteria or strains used in food fermentation (Table 1). Coryneform bacteria are industrially utilized for the production of Glu, and LAB strains (*Lactobacillus plantarum*, *Lactobacillus paracasei*, and *Lactococcus lactis*) are also able to synthesize Glu [51,52]. A study demonstrated that about 15% of the LAB strains isolated from Asian fermented foods are Glu producers [53].

From a functional viewpoint, the presence of a Glu-activated potassium channel was demonstrated only in the *Synechocystis* PCC 6803 strain, although, at least 100 prokaryotic channel proteins, containing putative Glu binding domains, have recently been identified [54]. Among these channels, 22 are homologs of vertebrate iGlu receptors [55]. Moreover, in the same way of eukarya, bacterial Glu is a substrate for GABA synthesis, via decarboxylation by Glu decarboxylase (GAD), which has been detected both in Gram-positive and Gram-negative bacteria [56,57]. 

These results allow hypothesizing that the use of bacteria producing Glu could represent a tool to modulate Glu signaling both locally and systemically. However, owing to the paucity of available information, at the moment, more efforts are needed to individuate microbiota bacteria able to produce, sense and respond to Glu.

### 4.3. Glutamate as a Neurotransmitter in the CNS and ENS

#### 4.3.1. Glutamate in the CNS 

Glu is the main excitatory neurotransmitter in the CNS, and both neurons and glial cells possess the molecular machinery responsible for regulating its synthesis, release and reuptake [64]. The Glu concentration in neuronal cytoplasm is ~5 mM, while astrocytic concentrations are lower (around 2–3 mM), however, Glu concentrations in cerebrospinal fluid or brain intercellular fluids range from 1 to 10 μM [65]. These concentrations are 5–50 fold lower than in the blood, giving rise to the intraparenchymal blood Glu concentration gradient, which depends on the ability of the blood brain barrier to prevent Glu entrance into the brain [66]. In the CNS, Glu is produced by neurons from transamination of α-ketoglutarate, originated in the tricarboxylic acid cycle, and from hydrolytic deamination of glutamine by phosphate-activated glutaminase [67]. Glu release from synaptic terminals is Ca^++^- and ATP-dependent and is under control of metabotropic autoreceptors and of several heteroreceptors [68]. Multimeric proton/Glu vesicular transporters (VGLUT1, VGLUT2, VGLUT3), transport Glu into vesicles for presynaptic storage: VGLUT1 and VGLUT2, are primarily expressed in glutamatergic neurons and in glial cells, VGLUT3, that has been detected in non-glutamatergic neuronal populations [64,68,69] (Table 2). Glu is actively removed from the synaptic cleft and transported into the cytosol against its concentration gradient, via excitatory amino acid transporters (EAAT). EAATs constitute a family of high-homology transmembrane proteins identified as EAAT1/GLAST, EAAT2/GLT-1, EAAT3/EAAC1, EAAT4 and EAAT5 [65,70] (Table 2). GLAST and GLT-1 are expressed prevalently by astrocytes and, to a minor extent, by neurons and endothelial cells in the brain. EAAC1 has a prevalent postsynaptic neuronal localization, while EAAT4 is highly expressed by Purkinje cells in the cerebellum, and EAAT5 is localized in the retina. EAAT1-3 play a crucial role in the regulation of intraparenchymal Glu [67]. Astrocytic cytosol is rich in glutamine synthase, which transforms uptaken Glu into glutamine. Once formed, astrocytic glutamine is transported into the extracellular fluid and is successively uptaken by neurons, where it is converted by the deaminase into Glu [71]. This homeostatic control of extracellular Glu prevents its accumulation with the consequent development of excitotoxicity [72]. 

#### 4.3.2. The Enteric Nervous System

The ENS is a complex network constituted of ganglia, interconnecting fiber strands and neuronal fibers innervating smooth muscle and epithelial cells, intrinsic blood vessels and gastroenteropancreatic endocrine cells from the esophagus to the anal sphincter [29]. The ENS controls different gastrointestinal functions such as motility, gastric secretion, transport of fluids across the epithelium, blood flow, nutrient absorption, in a rather autonomous way with respect to the CNS, and interacts with the immune and endocrine systems of the gut [73,74]. Gastrointestinal reflexes, such as propulsion of intraluminal contents (peristaltic reflex) or generation and progression of migrating myoelectric complexes (MMC) during prolonged interdigestive fasting periods are independent of the extrinsic innervation [73]. However, the ENS is not totally autonomous and the full neuronal control of the gastrointestinal functions derives from the integration of local reflexes, with reflexes mediated by sympathetic ganglia and afferent reflexes from the gut to the CNS, via vagal, splanchnic and pelvic nerves [29,75]. The ENS is composed of a large number of neurons, in humans 200–600 millions, the same number present in the spinal cord, which constitute three major plexuses: the subserous, the myenteric (located between the circular and longitudinal smooth muscle layers) and the submucosal (located in the homonymous layer) plexuses. At least 20 distinct types of neurons, classified according to their morphology, neurochemical coding, cell physiology, projections to targets and functional roles, constitute the enteric plexuses. Neurons in the ENS have been distinct in four major functional types: intrinsic primary afferent neurons, interneurons, excitatory and inhibitory motor neurons [73]. Intrinsic primary afferents are sensory neurons, which detect diverse stimuli (i.e., chemical and mechanical) in both the mucosa and muscularis propria and initiate appropriate motor, secretory and vasomotor reflex responses [74]. In different animal species, the chemical coding of primary afferent enteric neurons is highly conserved and is represented by cholinergic and serotoninergic neurons, peptidergic neurons containing tachykinins and calcitonin related gene peptide (CGRP) [74,76,77]. Excitatory and inhibitory motor neurons receive fast excitatory synaptic potentials and innervate the longitudinal and circular smooth muscle layers and the muscularis mucosae along the gastrointestinal tract [77]. The primary neurotransmitters for excitatory motor neurons are acetylcholine (ACh) and tachykinins. Several neurotransmitters have been identified in inhibitory motor neurons, including nitric oxide (NO), vasoactive intestinal peptide (VIP) and ATP-like transmitters, although NO is considered the primary transmitter [29,78,79]. Local intestinal reflexes are coordinated by several types of ascending and descending interneurons, which are characterized by different chemical coding, according to their projection and function [29]. A distinctive feature of the ENS is that enteric neurons communicate with different cell types, which constitute the enteric microenvironment including enteric glial cells, smooth muscle cells and the interstitial cells of Cajal, which are considered intestinal pacemaker cells, immunocytes of the gut-associated lymphoid tissue (which represent the most important immune cell reservoir of the human body), ECCs, which contain more than 20 identified hormones and microbes of the commensal flora [80,81,82,83]. In this latter context, there are several reports suggesting that the enteric microflora may influence the activity of both motor and sensory enteric neurons. Recently, chronic antibiotic treatment in juvenile mice induced complex morpho-functional neuromuscular rearrangements, determining a reduction in the gastrointestinal transit [81]. In adult mice, a two-week antibiotic treatment, inducing colonic dysbiosis, was associated with increased colonic contractility [84]. Specific bacterial strains may participate in maintaining normal intestinal motor function. GF rats displayed important abnormalities in the intestinal motor function characterized by significantly delayed intestinal transit and MMC period, which were partially reversed after colonization with either *Lactobacillus acidophilus* or *Bifidobacterium bifidum*. On reverse, colonization with *Escherichia coli* and *Micrococcus luteus* delayed gut motility [85]. The gut microbiota may also influence intrinsic primary neurons within the ENS. In an electrophysiological study, polysaccharide A, derived from *Bacteroides fragilis* was shown to stimulate myenteric plexus sensory neurons in vitro [86]. *Lactobacillus reuteri* increased excitability and the number of action potentials per depolarizing pulse decreased calcium-dependent potassium channel opening and decreased slow after-hyperpolarization in primary sensory neurons [87]. More recently, in GF mice, the electrophysiological properties of myenteric plexus primary afferent neurons were found to be altered, displaying reduced excitability that was restored after colonization with normal gut microbiota [88]. Microbial, as well as immune, factors appear to alter also the excitability of vagal afferent neurons that synapse with intrinsic primary afferent neurons [89]. For instance, components of *Lactobacillus rhamnosus* (JB-1) have a stimulant effect on vagal afferent neurons [90]. This microbe-driven effect on the vagus may favor a rapid communication of signals to the brain, unlike endocrine signaling, and may explain the positive effects of probiotics on brain function [91]. 

#### 4.3.3. Glutamatergic Enteric Neurons

Glu plays a role as a neurotransmitter in the ENS. The ability of luminal Glu to enter enteric ganglia, however, is uncertain, since ingested Glu was apparently unable to influence the gut neuromuscular function [92]. Similarly to the CNS, the presence of a functional blood barrier might prevent Glu entrance within enteric ganglia [93], where Glu is synthesized. In fact, in the ENS, the entire “Glutamatergic neurotransmitter machinery”, including vesicular and neuronal transporters and receptors, has been demonstrated in different animal species and in humans by means of histological, biomolecular and functional/pharmacological approaches [10]. Glu immunoreactivity within enteric neurons is concentrated in axonal terminals of the myenteric and submucosal plexus of different species, including rat, guinea pig and human [94,95]. In analogy with the CNS, myenteric neurons may synthesize Glu from glutamine hydrolysis [63,96], while co-localization of glutaminase and Glu was demonstrated in nerve bundles innervating the circular and longitudinal muscle layers of the rat stomach [97]. Glu is stored in varicosities within enteric neuron terminals, from which it is released in both a Ca^++^-dependent and Ca^++^-independent manner [98,99]. In the rat stomach and guinea pig ileum, immunohistochemical investigations showed the presence of Glu in intrinsic primary afferent neurons, suggesting that the amino acid may behave as a sensory co-transmitter within enteric neuronal circuitries, transmitting information from the mucosa to the ENS [94,100]. Electrophysiological studies support this hypothesis since both in the guinea pig ileum and colon depolarization of intrinsic primary afferent neurons was obtained after direct application of Glu or of its co-agonist, glycine, to interganglionic fibers [94,101]. Transporters controlling Glu re-uptake were identified in myenteric and submucosal neurons, glial cells and enterocytes in different animal models [10]. Abundant expression of GLAST/EAAT1 transporter was demonstrated on enteric glial cells of the mouse colon myenteric plexus [102], suggesting that enteric glia may contribute to maintain low extracellular Glu concentrations in the ENS, in analogy with astrocytes in the CNS [103]. In this context, morphological, molecular and functional studies showed the presence of glutamine synthase [104], Glu [95], iGlu receptors [105,106] in enteric glial cells. Glu transporters are also expressed on epithelial cells, as demonstrated by immunohistochemistry and in situ hybridization studies in the mouse small intestine, where they may participate to the initial steps of Glu metabolic pathways in the gut mucosa [107]. VGLUT are expressed in enteric neuron terminals of both intrinsic and extrinsic origin. In the small intestine, colon and rectum of different species, VGLUT2-immunoreactivity was found both in the submucosal and in the myenteric plexus of intrinsic primary afferent neurons [108,109]. Immunohistochemical approaches have shown the presence of VGLUT2 in the soma of a subset of NVG and DRG neurons, indicating that glutamatergic nerve terminals in the gut originate also from extrinsic vagal primary afferent neurons [108,109].

## 5. Glutamate Receptors: Distribution and Function along the Gut-Brain Axis

**Figure 2:**
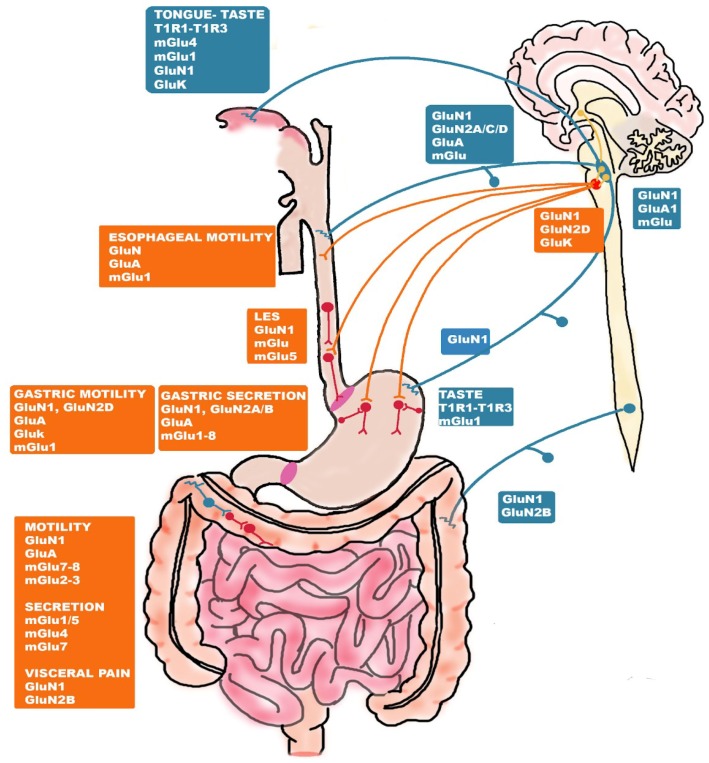
Distribution of glutamate receptors along the gut-brain axis. iGlu and mGlu receptors are located in ENS circuitries involved in the motor, secretory and sensory functions (motor neurons and interneurons in red and intrinsic primary neurons in blue). Glu receptors are present also on vagal and spinal extrinsic afferent pathways sending sensory information to the CNS (blue) and on effector pathways conveying excitatory and inhibitory inputs into the gastrointestinal tract from the CNS (orange). In the CNS, neurons (yellow) projecting from the hypothalamus to sensory vagal nuclei (nodose vagal ganglion, NVG, blue) in the brain stem and from the NVG to effector nuclei (dorsal motor nucleus, DMV, red) modulate digestive functions via activation of iGlu and mGlu receptors. Abbreviations: CNS, central nervous system; ENS, enteric nervous system (modified from Filpa et al., 2016 [10]).

Glu receptors are classified into two major types: ionotropic (iGlu) and metabotropic (mGlu) receptors. iGlu receptors, prevalently flux Na^+^ and Ca^++^ and are distinguished into three major subtypes, NMDA, α-amino-3-hydroxy-5-methyl-4-isoxazole propionic acid (AMPA) and kainate, according to their electrophysiological properties, sequence homologies and affinity for selective agonists. At a postsynaptic level, Glu activates AMPA and kainate receptors inducing a fast depolarizing response followed by a rapid decay, while NMDA receptors induce a more prolonged depolarization [110]. NMDA receptors are heterotetrameric proteins composed of two obligatory glycine-binding GluN1 subunits and two modulatory GluN2 (A–D) and GluN3 (A–B) subunits, which confer functional diversity to the receptor [111]. NMDA receptors are unique among the Glu receptor family in that the simultaneous binding of glycine to GluN1 and Glu to GluN2 is required for ion channel opening. A peculiarity of NMDA receptors is that, at rest, the ion pore is blocked by extracellular Mg^++^, and this blockade may be overcome by the depolarization induced by AMPA or kainate receptor activation [111]. AMPA and kainate receptors assemble as homo- or heteromers from four and five subunits, GluA1-4 and GluK1-5, respectively [110]. mGlu receptors belong to the superfamily of G-coupled receptor proteins, and have been subdivided into three major groups, Group I (distinguished in mGlu1 and mGlu5), Group II (mGlu2 and mGlu3) and Group III (mGlu4, 6, 7, 8) according to the homology of their molecular structure, pharmacological and physiological properties and related signal transduction pathways [112]. Group I receptors by coupling to phospholipase C, produce IP_3_, and the consequent release of Ca^++^ from intracellular stores, and diacylglycerol, to stimulate protein kinase C. Group II and III receptors reduce intracellular cAMP levels via adenylate cyclase inhibition. mGlu receptors participate to presynaptic regulation of Glu and of other neurotransmittes release [112]. Furthermore, mGlu receptors postsynaptically modulate the effects of Glu on neurons and glial cells [112]. All subtypes of iGlu and mGlu receptors have been localized to intrinsic and extrinsic neuronal circuitries involved in the regulation of sensory, secretory and motor functions along the gastrointestinal tract of different species, including rat, mouse, guinea pig and human [10,113] (Figure 2).

### 5.1. Glutamate Receptor-Mediated Taste and Savoriness

In the upper part of the gastrointestinal tract, Glu receptors play a fundamental role in transducing taste stimuli. Although dietary proteins do not, in general, elicit any specific taste sensations, Glu in free form elicits a unique taste distinct from sweet, salty, sour, and bitter, termed “umami” (one of the five basic tastes) [114]. Glu signaling in umami taste perception is conveyed to the rostral division of the NTS via facial, glossopharyngeal and vagus nerves and then further transmitted to the ventroposterior medial nucleus of the thalamus, from where thalamic afferents project to the primary gustatory cortex [115,116]. Several receptors that recognize and bind Glu are present on taste cells, comprising the heterodimer taste receptor type 1, member 1 (T1R1) and 3 (T1R3), which represents the best characterized of the Glu taste receptors. In mice, T1R1-T1R3 is activated by all L-amino acids, while in humans the heterodimer recognizes only Glu [117]. Zhao et al. (2003) [118] showed complete elimination of all responses to umami compounds in either T1R1 or T1R3 knockout mice. T1R1-T1R3 receptor allosterically binds other umami substances such as inosine 5’-monophosphate (IMP) and guanosine 5’-monophosphate (GMP), which, when present, strongly potentiate the umami taste. Particularly in the anterior tongue, mGlu receptors are involved in the translation of taste sensation [119,120]. mGlu4 and its truncated form, called “taste-mGlu4”, have been identified in taste buds [121]. Participation of mGlu4 in taste perception was confirmed by the reduction of the response to umami stimuli after both pharmacological blockades with specific mGlu4 antagonists and by genetic receptor deletion in mice [122,123]. mGlu1 and its N-terminal truncated form, called “taste-mGlu1”, have also been detected in taste buds by q-RT-PCR and immunohistochemistry [124]. Although mGlu1 knockout animal models have not yet been tested in taste experiments, a selective antagonist, 1-aminoindan-1,5-dicarboxylic acid, reduced responses to Glu in chorda tympani, a branch of the facial nerve, and in glossopharyngeal nerve, the two major taste nerves from the tongue [125]. In taste buds, Glu may also act as a neuromodulator by depolarizing taste cells at concentrations below those required for its detection as a taste stimulus [117]. Functional and molecular evidence suggest that Glu may also act as an “efferent” transmitter of taste sensation, via NMDA and kainate receptors located on taste cells, which may be involved in the modulation of taste signal before transmission to the brain [126,127]. In the stomach and small intestine epithelium, nutrient chemosensing cells, morphologically similar to taste cells, express T1R1-T1R3 and mGlu1 receptors and are able to transfer information of the luminal content, including Glu, to the brain, via the endocrine system and vagal pathways [128,129]. In anaesthetized rats, luminal IMP and Glu significantly increased vagal afferent nerve activity and induced autonomic reflexes involving activation of vagal celiac and splanchnic efferent nerves, strengthening the evidence that umami substances in the stomach send information to the brain via the vagus nerve [119]. Noninvasive functional magnetic resonance imaging studies on awake rats showed that intragastric MSG load administration significantly activates different brain regions such as the hippocampus and the amygdala, which are involved in memory, learning and emotion elaboration, as well as the dorsolateral hypothalamus and the medial preoptic area, involved in basic metabolism and body temperature regulation, respectively [115,130]. Vagotomy, strongly suppressed exogenous Glu-mediated effects in most forebrain regions.

### 5.2. Glutamate-Receptor Mediated Control of Esophageal and Gastric Function

In the esophagus, the control of propulsive activity as well as of lower esophageal sphincter (LES) are mainly controlled by vagovagal reflex pathways, although an intrinsic neuronal network is present [29]. Glu receptors may affect esophageal motility by modulating vagal afferents and efferent pathways and brainstem nuclei. The presence of different AMPA and NMDA receptor subunits has been demonstrated both peripherally and on vagal afferents projecting to brainstem regions involved in swallowing, such as central subnucleus of the NTS (NTSc) and in the compact formation of the nucleus ambiguous, by means of qRT-PCR, in situ hybridization, immunohistochemistry and functional approaches, as reviewed recently by Filpa et al. [10]. For example, in rat, both AMPA and NMDA receptors were involved in the activation of vagal esophageal afferents conveying excitatory inputs into NTSc after esophageal distension, underlying a reflex contractile response [131]. Interestingly, GluN1 and nNOS are co-expressed in second-order esophageal premotor neurons of the NTSc, which release NO in response to NMDA receptor activation [132]. This NMDA-mediated NO synthesis may have important implications on the esophageal motor function since NO is the primary neurotransmitter mediating vago-vagal inhibitory reflexes involved in esophageal propulsion and lower esophageal sphincter (LES) relaxation to allow food passage [73]. Glu participates to the control of LES pressure, by modulating the activity of peripheral nitrergic myenteric neurons as well as of neuronal circuitries in the DMV [133,134]. mGlu receptors may also participate to transmit vagal afferent signals from the esophagus to the CNS, and mRNAs and protein of all mGlu receptor types are expressed in the NVG and NTS of several species [135,136]. In addition, in the rat and human esophagus, mGlu1 and mGlu4 receptors are also located at a postjunctional level in the mucosal and smooth muscle layers [137,138]. Studies carried out in different animal models and in humans have demonstrated that compounds acting at NMDA, AMPA or mGlu receptors may represent useful tools to treat transient lower esophageal sphincter relaxations (TLESRs). TLESRs consist in prolonged distensions of the LES initiated by gastric distention in the absence of a swallow and are the major determinant of reflux in healthy subjects and in most patients with gastroesophageal reflux disease (GERD) [139,140]. The more convincing and promising results have been obtained from studies focusing on the ability of selective Group I, II and III agonists and antagonists to modulate TLESR, which allowed to identify mGlu5 as a major player [141,142]. In this view, small negative allosteric modulators of mGlu5, such as ADX10059, have been designed for the potential management of GERD [143,144]. Consistent with a role of Glu in the modulation of the sensory function in the gut, NMDA, AMPA and mGlu receptors are involved in esophageal pain perception and may participate to acid-induced esophageal hypersensitivity, suggesting a potential role for antagonist compounds for the treatment of patients with GERD, who display a low threshold for pain perception [10,145].

Similarly to the esophagus, in the stomach, the vagus nerve plays an important role in mediating gastric contractions and acid secretion, whereas the relevance of the ENS in the coordination of gastric functions is discussed [72]. Gastric glutamatergic pathways are mainly of extrinsic origin and may participate in either inhibitory or excitatory motor responses depending on the nature of the stimulus and the region involved [146]. In different animal models, gastric distension involved NMDA and AMPA/kainate receptor activation to transduce mechano- and chemosensitive vagal inputs onto vagal efferents, leading to either inhibition or excitation of gastric motility [147,148,149]. In the rat, retrograde tracing immunohistochemistry showed that all vagal afferent neurons projecting from the stomach to the NVG express GluN1, whereas GluN2C and GluN2D subunits were expressed by more restricted neuronal populations [150]. RT-PCR analysis revealed that all NMDA receptor subunits are present also within the stomach wall in both myenteric neurons and mucosal cells [151]. Pharmacological evidence of a potent local excitatory effect of NMDA and kainate receptors located on intrinsic myenteric neurons on the rat gastric fundus smooth muscle have also been given [152]. In the rat stomach, postjunctional GluN1, GluN2A and GluN2B, located on mucosal epithelial cells, submucosal and myenteric neurons, may influence histamine-induced acid secretion and blood flow [153]. mGlu receptors expressed by non-neuronal cells in the gastric mucosa, play a role in conveying sensory information to vagal afferent fibers and participate in digestion of food [115]. mGlu1-8 mRNAs were detected in different isolated cell fractions of the rat stomach, including parietal and chief cells, large and small endocrine cells, such as D cells of the rat stomach, which contribute to luminal Glu sensing as well as to the regulation of somatostatin secretion [154]. mGlu1 located on the apical membrane of the chief and parietal cells of the rat stomach participate to the gastric phase of protein digestion and mGluR2/3 receptors are involved in the control gastrin secretion and gastric acid production [155,156]. Glu, via mGlu receptors, may influence mucosal defense mechanisms to prevent subsequent injury attributable to excessive acid exposure in the duodenum. Thus, the pharmacological manipulation of intrinsic and extrinsic glutamatergic pathways impinging on the stomach may be exploited for the treatment of gastric acid hypersecretory disorders [137].

### 5.3. Glutamate-Receptor Mediated Control of the Intestinal Function

The ENS plays a major role in the control of intestinal secretory, sensory and motor functions [29]. Different iGlu and mGlu receptors are abundantly expressed in the ENS innervating the small and large intestine of different species [10]. Immunohistochemistry and in situ hybridization studies revealed that GluN1 is abundantly expressed in intestinal submucosal and myenteric neurons in humans and in other species, reflecting the functional relevance of this receptor pathway within enteric ganglia [94,95,105]. In both small and large intestine myenteric plexus, activation of NMDA receptors enhances contractile cholinergic responses indirectly by interacting with nitrergic pathways [157,158]. GluA2/3 and GluA4 subunits have also been detected in cholinergic and non-cholinergic interneurons and enteric motor neurons in different animal models, and the ability of AMPA receptors to increase both spontaneous and electrically-evoked contractions of the colon has been demonstrated in the guinea pig colon and mouse small intestine [10,102]. In the submucosal and myenteric plexus and in nerve fibers of the rat and guinea pig small intestine, the presence of Group I, Group II and Group III receptors was demonstrated by immunohistochemical, biomolecular, electrophysiological and functional studies [10]. In particular, mGlu7 and mGlu8 are abundantly expressed in human, rat, mouse and guinea small intestine myenteric plexus [159,160,161]. In the guinea pig, mGlu8 agonists induced a facilitatory effect on motility, which was blocked by specific antagonists, suggesting the occurrence of a tonic glutamatergic control of colonic motor responses via mGlu8 receptors [159]. In the human colon, a role for mGluRs in the control of colon peristalsis and electrolyte transport has been proposed [160]. Colonic mGlu receptors may participate in Glu mediated modulation of the intestinal mucosal function by acting either on enteric neurons or on non-neuronal epithelial cells. Interestingly, exposure of mucosa/submucosa preparations to a selective mGlu7 agonist, AMN082, potentiated stress-induced secretory responses suggesting a role for mGlu7 receptors in the development of stress-associated gastrointestinal secretory disorders such as diarrhea or constipation [161]. Glu receptors, mainly NMDA receptors, participate also in transmission of visceral sensitivity from the small and large intestine. In the rat, GluN1 immunoreactivity was found in the soma of extrinsic primary afferent thoracolumbar DRGs, as well as in their peripheral terminals innervating the colonic mucosa [162]. GluN1 subunit is largely co-expressed with capsaicin-sensitive transient potential vanilloid receptor, TRPV-1, which are involved in the neurotransmission of visceral pain [162]. Peripherally and centrally located NMDA receptors may contribute to the development of visceral hypersensitivity in non-pathological conditions. Both intrathecal and intraperitoneal administration of MK-801, a non- competitive NMDA antagonist, completely abolished hypersensitivity responses to both innocuous (low pressure) and noxious (high pressure) stimuli induced by colorectal distension in rats [163]. 

## 6. Glutamatergic Dysfunction along the Microbiota-Gut-Brain Axis: Relation to IBS and IBD 

**Figure 3:**
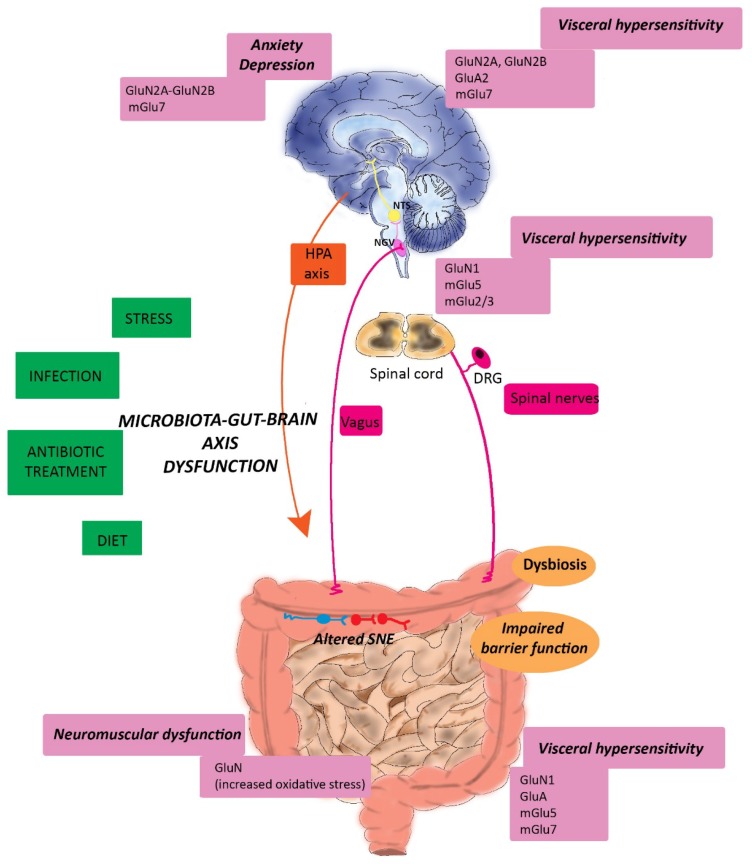
Glutamate receptors and derangement of the microbiota-gut-brain axis. Several factors, including stress, previous infection, antibiotic treatment and diet may influence the stability of the microbiota-gut-brain axis. Derangement of this bi-directional communication axis may underlay the development of major symptoms involved in the pathogenesis of gastrointestinal diseases such as IBS and IBD, including visceral pain, altered motor function and CNS disorders, such as anxiety and depression. Glu participates to development of these symptoms by activating both iGlu and mGlu receptors located peripherally, in the gut, in an intermediate station (spinal cord and brainstem) and in higher centers of the gut-brain axis. Abbreviations: DRG, dorsal root ganglion; NGV, nodose vagal ganglion; NTS, nucleus of the solitary tract; ENS, enteric nervous system, HPA, hypothalamic-pituitary axis.

Alterations of the glutamatergic neurotransmission represent key pathogenetic factors contributing to the development of several CNS diseases [9]. Derangements of glutamatergic enteric pathways may also influence the progression of gut disorders [10]. Changes in Glu receptor expression and function are principally involved in glutamatergic transmission changes, which may dispose of more severe conditions underlying neurotoxic accumulation of extracellular Glu concentrations (excitotoxicity). Glu-mediated excitotoxicity causes excessive postsynaptic excitation, resulting from the enhanced pre-synaptic release of the amino acid, superimposed on deficient uptake and/or cytosolic efflux [9,164]. Glu-mediated excitotoxic cellular damage is primed by an excessive rise in intracytoplasmic Ca^++^ concentrations, mediated by extra-synaptically located NMDA receptors and by non-NMDA receptors, leading to neuronal necrosis and apoptosis and death [164]. Analogously to the CNS, overactivation of iGlu receptors on enteric neurons induced excitotoxicity, which was associated with intestinal ischemia/reperfusion injury and chronic inflammation [9,41,99,105]. Although enteric ganglia are normally impermeable to luminal Glu, we cannot exclude that, in pathological conditions, such as in inflammatory bowel disease (IBD) or in irritable bowel syndrome (IBS), all characterized by enteric neuropathies, permeability to the amino acid increases, leading to altered neuronal responses locally and along the microbiota-gut-brain axis. It is now clear that integrated actions and communication between the microbiota, the ENS, the ANS and the CNS may sustain the development and perpetuation, not only of ENS disorders but also of CNS diseases [3]. Numerous evidence suggests, indeed, that dysfunctions of the microbiota-gut-brain axis may underlay the development of CNS diseases, such as neuropsychiatric, neurodevelopmental and cognitive neurodegenerative dysfunctions [2]. Although of high clinical interest, the involvement of the microbiota-gut-brain axis in the development of major CNS diseases has been elegantly reviewed elsewhere and a comprehensive evaluation of this relationship goes beyond the scope of this review [4,5,6]. In this review we will focus on the most clear-cut evidence of the possible involvement of glutamatergic transmission derangement along the microbiota-gut brain axis and the development of IBS and IBD, both characterized by elevated psychiatric co-morbidity [13,14] (Figure 3)

### 6.1. Irritable Bowel Syndrome

IBS represents the most frequent among the functional diseases of the gastrointestinal tract, with a prevalence of the 10%–15% worldwide, mainly involving patients with less than 50 years of age and with a ratio 2:1 between females and males [165]. IBS is a multifunctional chronic or recurrent disorder, with main symptoms entailing abdominal pain and distension, associated with altered bowel habits and disordered defecation, underlying either constipation (IBS-C) or diarrhea (IBS-D) or both [13]. Different factors, including, abnormal motility, changes in CNS processing of visceral hyperalgesia, ANS dysfunctions, familiarity, psychosocial triggers and postinfectious events concur to the development of symptoms, although the exact etiopathogenesis remains unknown [13,165]. A widely accepted view is that IBS represents a microbiota-gut-brain disorder [13,35,166]. The correlation between IBS development and a previous bacterial infection is highly suggestive of a relationship connecting changes in the gut microbiota composition with the risk to develop IBS [166,167,168]. Almost 10% of patients affected with IBS refers to the first symptoms after an episode of bacterial gastroenteritis, the so-called post-infectious-IBS (PI-IBS). The risk to develop a PI-IBS following an enteric infection is between 3%–36% and the incidence seems to be correlated to the pathogen microorganism underlying the disease, the highest incidence (36%) being associated with *Campylobacter jejuni* and *Escherichia coli* O157:H7 [169]. In PI-IBS patients, changes in both sensory and motor responses may depend, at least in part, upon a subclinical low-grade immune activation, in the absence of overt features of organic pathological inflammation. In intestinal biopsies of IBS patients, the number of immunocytes augments and, seemingly, serum levels of pro-inflammatory cytokines, including IL-6, IL-8, TNFα and IL-1β, and of the neutrophil marker, fecal calprotectin, increase with respect to healthy controls [170,171,172]. In addition, colonic biopsies from IBS patients show enhanced expression of toll-like receptors (TLRs) [173,174]. TLRs are members of the pattern recognition receptor (PRR) family that play a central role in the innate immune response, by recognizing pathogen-associated molecular patterns (PAMPs) and transduce the signals required for an effective innate immune response [175]. Several metagenomic studies, carried out in recent years correlate changes in the saprophytic flora composition with the development of IBS symptoms, showing that IBS patients may present a situation of dysbiosis with respect to healthy subjects [13,35,166]. Although some reports have been provided with conflicting results, depending on the subgroups of IBS patients considered, a consensus emerged at the phylum level suggesting an increased ratio of the Firmicutes to Bacteroidetes [176,177]. 

#### 6.1.1. Glutamatergic Transmission and IBS-associated Visceral Pain

Numerous preclinical and clinical evidence suggest that changes in the microbial flora are correlated with the development of visceral hypersensitivity, which represents one of the major symptoms in IBS patients and consists of a diffuse and poorly localized chronic abdominal pain [75]. In GF mice the excitability of myenteric primary afferent neurons was altered with respect to control and was restored after colonization, suggesting that a normal saprophytic flora is essential for the activity of intrinsic sensory neurons in the gut [88]. This effect extends to the extrinsic sensory innervation since in mice, administration of live *Lactobacillus reuteri* (DSM 17938) reduced jejunal spinal nerve firing evoked by gut distension with an intraluminal balloon or capsaicin [178]. In IBS patients, an increased amount of Proteobacteria has been correlated with the scores of visceral pain [176,179]. Transplantation of fecal microbiota from IBS-C patients, experiencing visceral hypersensitivity to colorectal distension, to GF rats increased visceral sensitivity with respect to rats transplanted with fecal material from healthy volunteers [180]. Antibiotic treatment during postnatal development induced visceral hypersensitivity in adult male rats [181], while probiotic treatment ameliorated these symptoms [182]. Disturbance of the gut microbiota in adult mice induced local changes in immune responses and enhanced visceral pain signaling [183,184]. These latter studies suggest the existence of a strong correlation between dysbiosis occurring in both early life or during adult age and development of visceral pain responses [35]. The ability of NMDA receptor antagonists to reduce pelvic and splanchnic afferent stimulation after application of mechanical stimuli in the colon, suggest the participation of endogenous glutamate in the modulation of mechanosensitive pathways [185]. In rats, intrathecal administration of NMDA in the spinal cord, concentration-dependently enhanced visceromotor responses to noxious colorectal distension and this effect was blocked by the NMDA receptor antagonist, (-)-AP5 and by the antagonist at the glycine site associated with NMDA receptor, 7-chloro-kynurenic acid, a derivative of KynA [186,187]. The local release of neuropeptides, such as CGRP and SP from rat DRG cell bodies and from peripheral terminals of primary afferent innervating the colon, involved in neuroinflammation responses and hyperalgesia, was mediated by NMDA receptor activation [162,188,189,190]. A major role for GluN2B was evidenced in the development of visceral pain symptoms after experimentally-induced colitis with trinitrobenzesulfonic acid (TNBS)-induced colitis in rats [191]. In the same animal species, visceral hypersensitivity, developing after administration of mustard oil, enhanced the expression of GluN2B and GluA2 receptors in the anterior cingulated cortex neurons, a brain region critically involved in the modulation of visceral pain responses [192]. The predominance of either GluN2A or GluN2B has important influences on NMDA receptor function since GluN2A containing receptors are considered to have neuroprotective actions, while GluN2B subunits are coupled to neurotoxicity [193]. Preclinical studies resorting to rodent models of IBS, associated with the development of dysbiosis, evidenced alterations of iGlu receptor expression, both locally and in the CNS. In a rat model of PI-IBS, obtained after oral administration of *Trichinella spiralis* larvae, the expression of GluN1 and AMPA receptors, postsynaptic density-95, synaptophysin and glial-derived nerve growth factor, was up-regulated in the rat ileum, caecum and colon, 8 weeks post-infection, suggesting the occurrence of neuroplasticity [194]. Recently, in a rat model of maternal separation-induced IBS associated with alterations of the microbiota homeostasis, bilateral hippocampal injection of the AMPA receptor antagonist, CNQX, reduced visceral pain perception to colorectal distension. In addition, GluA2 receptor levels significantly increased in the hippocampus of IBS-like rats, with respect to controls, both in normal conditions and after high electrical field induced LTP-responses, suggesting a possible involvement of GluA2 subunits in central mechanisms of chronic visceral pain control [195]. Persistence of pain perception in IBS, depends upon changes in afferent neurons and CNS pain processing pathways, leading to chronic visceral hypersensitivity [75]. In this context, NMDA receptors in the spinal cord play an important role, favoring the integration of complex neuronal networks to amplify nociceptive signals, thus inducing the “wind-up” of central responses to nociceptive stimuli [75]. Symptoms of visceral pain were observed in rats, sixteen weeks after cessation of TNBS treatment, without evident signs of colitis [196]. In successive studies of the same group, hyperalgesia after cessation of a TNBS treatment was associated with GluN1 expression up-regulation in the spinal cord and in the myenteric plexus [197,198]. mGlu receptors located in the ENS, on spinal primary afferents and at supraspinal sites, are also implicated in visceral pain perception and development of visceral hyperalgesia. For example, blockade of mGlu5 with the selective antagonist MTEP inhibited responses to colorectal distension of rat pelvic mechanoceptor afferents in vitro [199]. In the same study, in vivo intravenous injection of MTEP and of another selective mGlu5 antagonist, MPEP, inhibited viscero-motor responses and cardiovascular changes after colorectal distension in conscious rats [199]. The Authors postulated that mGlu5 receptors involved in mechanically-evoked visceral nociception in the gut are located peripherally, on nerve endings of colorectal afferents, although the participation of centrally located mGlu5 receptors could not be excluded since both agents are characterized by high brain permeability. In another study, colonic noxious stimulation enhanced c-fos positive neurons in the rat DRG of the thoracic and lumbar spinal cord, which was significantly reduced by MPEP [200]. In a mouse model of colitis carrying IL-10 gene deletion, a drastic reduction of mGlu5 receptor expression on enteric glial cells has been suggested as a possible protective mechanism to limit glial mGlu5 receptor-mediated stimulation of NMDA receptor and development of toxicity [201]. Johnson et al. [202] have recently demonstrated that, in vivo oral administration of the prodrug LY2969822, which rapidly converts to the brain penetrant, potent and subtype-selective mGlu2/3 receptor agonist, LY2934747, reduces pain behaviors across a broad range of preclinical pain models, including inhibition of nociceptive response to colorectal distension in normal and sensitized rats with acetic acid. Both painful responses and sensitization involve blockade of mGlu_2/3_ receptor-mediated activation of nociceptive neurons in the spinal cord. In a stress-sensitive Wistar Kyoto rat strain, which spontaneously exhibits visceral hypersensitivity as well as anxiety-like behaviors, the potent, selective and brain permeant mGlu7 negative allosteric modulator, ADX71743, normalized visceral hypersensitivity and reduced stress-induced anxiety-like behavior by modulating both centrally and peripherally located mGlu7 receptors [203]. In contrast with the availability of promising mGlu antagonists for GERD treatment, the number of translational studies evaluating mGlu receptors as potential targets in the management of visceral pain is, however, low and, to our knowledge, there are no published clinical trials, at the moment. Regulation of Glu transport may also be involved in pain perception and modulators of Glu re-uptake may be more efficacious and safer than modulators of ionotropic Glu receptors, due to the negative side effects induced during long-term pain treatment [203]. Inhibition of EAAT by intrathecal administration of dl-threo-b-benzyloxyaspartate (TBOA), induced visceral pain in rats [204]. The systemic administration of riluzole, an activator of Glu transport via EAAT, counteracted gastrointestinal hypersensitivity in rat and human models of visceral hypersensitivity [205,206]. Expression of EAAT-1 diminished in the lumbar region of the spinal cord in a maternal-separation model of visceral hypersensitivity, moreover, activation of EAAT2, the main glial transporter for Glu re-uptake, was protective against visceral pain [203,204]. In mice, overexpression of EAAT2, either after genetic manipulation or after treatment with the beta-lactam antibiotic, ceftriaxone, induced a protective effect against colonic distension-induced nociception [207,208]. Although the exact mechanism of ceftriaxone-mediated modulation of EAAT2 has not yet fully discovered, the hypothesis that its antimicrobial activity may bear consequences on Glu homeostasis along the microbiota-gut-brain axis pathways involved in visceral pain cannot be excluded [166]. 

#### 6.1.2. Glutamatergic Transmission and IBS-associated Psychiatric Disorders

IBS patients commonly experience psychiatric disorders, and a recent meta-analysis study shows that anxiety and depression levels are significantly higher in IBS patients vs healthy volunteers, regardless of IBS-subtype [209]. Both major depressive disorders and anxiety disorders are considered as the most frequent stress-related disorders [210]. Numerous evidence, indeed, suggests that IBS symptoms may be induced or enhanced by stressor stimuli [35]. Stress is considered as a dynamic process in which physical and/or mental homeostasis is triggered by both exogenous and endogenous stressors. The outcome to stressor stimuli depends on the type of stimulus and its severity, the time of exposure and the susceptibility/resilience of the organism [211]. The gut microbiota plays a fundamental role in the regulation of the host microbiota-gut-brain axis activation in response to stressor stimuli [212]. As suggested by studies carried out on GF rodents, after antibiotic and probiotic treatments, this modulatory function involves activation of hypothalamic-pituitary adrenal (HPA) axis, as well as the induction of immune and neuroendocrine responses [213,214,215,216] (Figure 3). For example, in a seminal study on GF mice, a mild stress restraint induced elevation of corticosterone and ACTH plasma levels, which were reversed by specific colonization with Bifidobacteria species [213]. These observations have been confirmed by successive preclinical studies showing that probiotic treatment may normalize HPA axis dysfunction induced by stress in early-life [214]. There is however a bidirectional microbial-neuroendocrine relationship, since stress may have long-term effects on the microbiota composition, as demonstrated both in early-life and adulthood [212]. Cortisol secreted after stress-induced HPA activation can affect immune cells and cytokine secretion both systemically and in the gut. This latter local effect may alter gut permeability and barrier function, and, consequently, the gut microbiota homeostasis and composition [1,30,49]. Indeed, prolonged exposure to stress causes ultrastructural alterations of the intestinal barrier, which, coupled to changes in the microbiota composition, may favor systemic translocation of different bacteria strains, such as Lactobacillus spp. and activation of an immune response [217,218]. Involvement of the innate immune system, favors the development of a proinflammatory state and secretion of intestinal secretory IgA, impacting on intestinal homeostasis and eventually reinforcing a dysbiosis [219]. In addition, stress-related mediators and neurotransmitters, such as catecholamines, may facilitate the growth of bacteria, such as isolated strains of non-pathogenic *E. Coli* as well as pathogenic strains such as *Escherichia coli* 0157:H7 [220]. In the CNS, a neuro-immune response develops, leading to TLRs-mediated neuroinflammation, which is prevented by antibiotic treatment [221]. In these conditions, the homeostasis of other neurotransmitter and neuromodulator pathways, including glutamatergic pathways, in brain regions such as hippocampus, amygdala and cingulate cortex, involved in stress responses may change [6]. Indeed, the participation of glutamatergic transmission to stress-related responses in different CNS regions has been shown resorting to several animal models and principally involves dysregulation of NMDA, AMPA, mGlu2/3, mGlu5 and, as recently proposed, mGlu7 receptors [222,223,224]. Data from both animal and human studies indicate that NMDA receptors play a fundamental role, since their blockade does not only reduce the negative impact of stress but may have anxiolytic and antidepressant effects [225,226]. Interestingly, ketamine, an antagonist at NMDA receptors, and more recently, the partial antagonist at the glycine site associated with NMDA receptor, GLYX-13, provided rapid onset of antidepressant effects, possibly caused by increased neuroplasticity involving AMPA receptors [227,228]. Stress-related perturbations of the microbiota-gut-brain axis may have important consequences on the expression and activity of NMDA receptors as well as on brain-derived neurotrophic factor (BDNF), a neurotrophin fundamental for neuroplasticity, whose function is strictly correlated to NMDA receptor activation in different CNS regions [229,230,231,232,233,234]. Interestingly, the fast antidepressant effect of both ketamine and GLYX-13 requires BDNF and molecular pathways downstream to its high-affinity receptor TrKB [230]. Corticosterone-induced postnatal stress in young-adult BDNF heterozygous mice was associated with downregulation of BDNF expression and dysregulation of NMDA receptor subunit expression in the hippocampus [230]. BDNF and TrKB levels in the CNS are influenced by the gut microbiota composition [213,235]. For example, BDNF levels were lower in the hippocampus of GF mice as well as in mice undergoing massive antibiotic treatment to induce dysbiosis, compared to controls [213,235] Interestingly, colonization of GF mice with fecal matter from SPF mice or probiotic administration, resulted in partial and complete normalization of anxiety-like behavior as well as of the BDNF levels [213]. Altered BDNF levels in the hippocampus of GF mice were associated with a decreased expression of GluN2A subunit compared to controls [213]. Clarke et al. (2013) [233] found that male, but not female, GF mice displayed reduced BDNF mRNA levels of expression in the hippocampus. However, in another study, only in the hippocampus of GF female mice, BDNF levels increased and such enhancement was associated with decreased GluN2B levels and anxiety-like behavior [234]. Overall these observations suggest that microbiota-induced alterations in CNS neurochemicals may be gender-specific [236,237]. Interestingly, administration of fructo-oligosaccharide (FOS) and galacto-oligosaccharide (GOS) prebiotics to rats was associated with increased levels of expression BDNF and of the GluN1 subunits in the hippocampus gyrus dentate [238]. Analogously, a combination of FOS and GOS showing a beneficial effect on stress-related behaviors, elevated BDNF mRNA in the mice hippocampus, while administration of B-GOS in rats had pro-cognitive effects, involving upregulation of cortical NMDA receptors [239,240]. Development of depressive mood disorders represents a further manifestation of stress-induced disorders observed in IBS patients and is correlated to gut microbiota dysbiosis [35]. In psychiatric patients with major depressive disorders, changes in gut microbiota have been investigated with different outcomes concerning the phylum Bacteroidetes, since some studies indicate a decrease [241] or an increase [242,243] of their abundance. The correlation between changes in gut microbiota composition and depression has been suggested also from preclinical studies showing that transplantation of fecal microbiota from depressed patients to GF or dysbiotic rodents induced a depressive-like phenotype in the animals [241,244]. The decrease in Bacteroidetes levels observed in IBS patients was correlated with the development of depression and anxiety [176,177,241] and reflected gut microbial composition changes observed in some patients with major depression [176,241,242,243]. Emerging evidence suggests that the diversion of the tryptophan metabolism from the 5-HT pathway towards the Kyn pathway may have an important role in the manifestation of psychiatric disorders such as anxiety and major depression [37]. In GF mice, induction of depressive mood after fecal transplantation was associated with an increase in the Kyn/tryptophan ratio [2]. Changes in tryptophan metabolism have been correlated with the manifestation of depressive symptoms also in IBS patients [245]. The activity of IDO, the immune sensitive enzyme responsible for tryptophan degradation enhanced in IBS patients, while the levels of the neuroprotective KynA and the ratio between KynA/Kyn decreased [246]. In spite, of the limited number of patients selected, a significant correlation was observed between the decrease of KynA and 5-HT in duodenal mucosal biopsy specimens and the psychological index state of IBS patients [245]. These observations suggest that modulation of the Kyn/tryptophan pathway, possibly influencing NMDA receptors in CNS regions involved in the development of depression [9,37], may provide useful therapeutic tools to prevent and /or reduce psychiatric co-morbid manifestations of IBS.

### 6.2. Inflammatory Bowel Disorders

IBD primarily comprises two diagnostically distinct, but pathologically similar disorders: Crohn’s disease (CD), and ulcerative colitis (UC), with increasing incidence worldwide [247]. CD is characterized by an inflammatory response developing along the gastrointestinal tract, while inflammation in UC is restricted to the rectum and colon [84]. Inflammation develops as a consequence of an exaggerated immune response to luminal antigens derived from the gut microbiota or from infecting pathogens in genetically predisposed individuals, although the exact etiology is unknown [248]. Patients with IBD commonly manifest symptoms suggestive of disturbed gastrointestinal function, characterized by sensory, motor and secretory alterations [249]. IBD is associated with profound transient alterations of the whole intestinal, such as prominent mucosal damage, abnormal secretion and visceral sensation. Long-term changes involving enteric neurons and the smooth muscle layers may lead to persistent dysmotility. Inflammation leads to derangements of enteric neuronal circuitries with increased neuronal hyperexcitability of primary afferent neurons, synaptic facilitation and reduced inhibitory neuromuscular neurotransmission [250,251]. The cross-talk occurring among different cell populations, constituting the enteric microenvironment, and infiltrating inflammatory cells may account for the structural and functional changes occurring in enteric circuitries in response to an inflammatory stimulus [252]. Neuronal cells in the ENS are located in close proximity to mucosal immunocytes and may regulate one another’s functions by releasing a complex set of cytokines, neurotransmitters and hormones. Neuronal activation can lead to degranulation of mast cells and the recruitment of neutrophils to the area. Furthermore, neuropeptides, such as SP and VIP, released by enteric nerves, may activate their receptors located on immune cells, inducing immunocyte differentiation and influencing IgA production [184]. The enteric microbiota represents a further fundamental player in the development of IBD, influencing inflammation-induced enteric neuron derangement. The importance of the gut microbiota in IBD development has emerged on the basis of clinical studies showing that IBD patients often develop dysbiosis, and that, some antibiotics are efficacious in the prevention and treatment of inflammation both in humans and in animal models [249]. Metagenomic studies have shown qualitative and quantitative differences in the microbiota composition in IBD patients, which are mainly characterized by a reduced fecal amount of the phylum Firmicutes (especially *Faecalibacterium prausnitzii*) and increased levels of the phylum Proteobacteria, including *E. coli*, with respect to healthy individuals [253,254]. The existence of specific pathobionts, i.e., commensal microorganisms, that under specific environmental or genetic conditions, can cause the disease, however, has not yet been demonstrated, although, elevated levels of a particularly invasive adhesive strain of *E. coli* (AIEC) have been found in biopsies of patients with active CD [184,255]. The correlation between dysbiosis and IBD has been demonstrated also in preclinical models of genetically modified mice, which spontaneously develop the disease. For example, in mouse models either overexpressing IL23 or carrying a deletion for IL10, which play a pro-inflammatory and anti-inflammatory action via activation of Th17 and Treg lymphocytes, respectively, spontaneous colitis developed only in the presence of a healthy microbiota [249,256]. Another important issue suggesting the crucial role of the gut microbiota in IBD pathogenesis derives from the observation that microbial components may be detected in the inflammatory lesions [3,248]. Interestingly, genome-wide association studies suggest that TLRs are implicated in IBD pathogenesis [257,258]. A consistent number of mutant mouse models have linked TLR signaling activation by microbial metabolites with epithelial repair and homeostasis after experimentally-induced colitis, showing that TLRs play a protective role on intestinal epithelium, by promoting epithelial cell survival, inhibiting apoptosis and recruiting stromal and myeloid cells [60,259,260]. In addition, both TLR2 and TLR4 are also present on neurons in the ENS, and TLR2s may regulate intestinal inflammation by controlling ENS structural and functional integrity [261,262]. In this scenario, it is particularly important to discover possible neuromodulators involved in the pro-inflammatory states, in order to prevent the occurrence of more obvious inflammatory conditions. 

#### 6.2.1. Glutamatergic Transmission and IBD-associated Neuromuscular Dysfunction

Glutamatergic transmission may sustain the development of neuroinflammatory responses in the gut, involving the modulation of oxidative and nitrosative stress pathways [41]. Glu, via NMDA receptor activation and subsequent elevation of intracellular free [Ca^2+^] may lead to the generation of peroxynitrite, derived from superoxide formation, via xanthine oxidoreductase (XOR), and NO generation by NOS [41]. In addition, radicals generated during NMDA-stimulated metabolism of arachidonic acid may sustain oxidative injury [263]. In this latter context, clinical studies have shown that in pro-inflammatory conditions, both NO and prostaglandin may represent important factors for gastrointestinal motility inhibition [264]. In this view, an antagonist to NMDA receptors may be neuroprotective in the course of gut inflammation and Glu-induced neurotoxicity, suggesting a possible role of endogenous Glu in both acute and chronic inflammatory conditions. Interestingly, evidence obtained in animal and human studies has pointed to the possible efficacy of KynA, an endogenous NMDA receptor antagonist, and its derivatives in IBD management [41]. In the dog and rat colon, administration of KynA during the acute phase of an experimentally-induced inflammation decreased motility index, NO and ROS production and, consequently, peroxynitrite formation [41,265,266]. Elevated KynA serum levels were found in patients with either IBD or coeliac disease [267,268]. This spontaneous modulation of the Kyn pathway in favor of KynA production may represent a neuroprotective approach, to compensate for inflammation-induced increased NMDA receptors activity contributing to derangement of the gut neuromuscular compartment and deserves attention by future investigations. Perturbation of tryptophan metabolism inducing elevated Kyn levels during gut inflammation depends upon stimulation of IDO by several factors such as cytokines, cortisol, but also by the perturbed microbiota [269,270,271].

The duration, extent and intensity of the inflammatory challenge (acute vs chronic) may strongly influence the response of enteric neuronal circuitries [253]. Long lasting and severe inflammation is associated with alterations of NO transmission, and consequent changes of smooth contractility, as well as with increased production of different pro-inflammatory cytokines, including IL-1β, IL-6 and TNFα [272]. In these conditions, overactivation of NMDA receptors determined neurotoxic death via excessive NO production [41]. In a rat model of TNBS-induced chronic colitis, KynA and SZR-72, a centrally-acting KynA analogous, normalized the increased frequency of bowel movements, reduced the NO-linked nitrosative stress as well as IL-6 and TNFα production [273]. Accordingly, in the rat intestine, NMDA receptor activation was associated with the production of pro-inflammatory cytokines, such as TNF-α, suggesting the existence of a possible NMDA-induced modulation of a neuroinflammatory response in the ENS, as demonstrated in the CNS [274]. Overall these data suggest that Glu may play an important role in the transduction of local inflammatory signals influencing gastrointestinal motility. In particular, modulation of the glycine site associated with NMDA receptors may represent a promising therapeutic target to treat neuromuscular dysfunctions associated with IBD. 

#### 6.2.2. Glutamatergic Transmission and IBD-associated Psychiatric Disturbances

Stress-related disorders such as major depression and generalized anxiety, are more common in IBD patients than in controls and may influence disease prognosis as well as the treatment outcome [14,275]. These psychiatric disturbances may be related to alterations in the saprophytic microflora homeostasis. Induction of experimental colitis in mice with dextran-dodecilysulfate (DSS) was associated with anxiety behaviors and cognitive deficits, which were prevented by a probiotic *(Lactobacillus rhamnosus* R0011 and *Lactobacillus helveticus* R0052), suggesting the involvement of the gut microbiota on behavioral disturbances associated with intestinal inflammation [276]. In rats, the increase in anxiety- and depression-like behavior accompanying experimentally-induced colitis was associated with increased firing rates of colonic afferents and may be evidenced only when vagal afferents are intact, indicating that these behavioral changes were principally neurally-mediated [277]. However, the same group showed in another study that an experimentally-induced inflammation in mice by administration of the non-invasive parasite, *Trichuris muris*, induces psychological disturbances probably via non-neuronal, immune pathways since anxiety-like behaviors were not prevented by vagotomy [278]. In this latter study, development of a low-grade inflammation was associated with an increase of circulating Kyn levels, enhanced Kyn/tryptophan ratio, and decreased hippocampal BDNF mRNA [278]. Interestingly, the anti-inflammatory etanercept and, to a lesser extent, budesonide, which are currently used in IBD therapy, reduced Kyn levels and normalized behavior [278]. These observations suggest that the unbalanced Kyn/tryptophan ratio and the shift of the tryptophan metabolism from the 5-HT synthesis to Kyn and its downstream metabolites may underlay psychological perturbations observed in IBD. Enteric 5-HT plays a key role in the modulation of several gut functions, including sensory, secretory and motor function, while in the CNS, 5HT is involved in the modulation of mood, behavior and cognitive functions [279]. Thus, inflammation-induced disruption of 5-HT homeostasis may participate in both dysmotility and development of mood disorders in IBD patients [279,280]. As previously mentioned, gut inflammation enhances plasma levels of Kyn, which by crossing the blood brain barrier, is transformed in the brain into its metabolites, principally KynA and quinolinic acid [279]. Quinolinic, a neurotoxic agent, acting as an agonist at NMDA receptors may participate in the development of depression [280]. Data from clinical investigations point to a role of the KynA/quinolinic acid ratio as an index of neuroprotection, and a reduced ratio is indicative of possible inflammation-induced depressive disorders [281]. Overall these observations suggest that modulation of NMDA receptor activation may represent a unifying mechanism linking the glutamatergic hypothesis of inflammation-induced depression and dysbiosis [282].

## 7. Perspective: Areas of Importance for Advancing the Field

The studies reported in the present dissertation suggest that modulation of ionotropic and metabotropic receptors along the microbiota-gut-brain axis may influence both gut and brain homeostasis. However, glutamatergic mechanisms influencing the activity of neuronal circuitries along this bidirectional communication axis, in normal and disease states, remain largely to be clarified. 

Future investigations would benefit from experimental approaches aiming to better characterize the functional properties of different Glu transporters and Glu receptor subtypes on specific neuron types along the microbiota-gut-brain axis. In this view, it would be of interest to resort to transgenic animal models, carrying deletion for Glu receptor subtypes and transporter systems, to target the expression/deletion of specific receptor subunits as well as to apply elective single-cell patch clamp techniques to measure the electrophysiological properties of Glu receptors subtypes in specific enteric neuronal populations. The majority of preclinical and clinical studies, up to now, have been used iGluR antagonists, whose potential clinical usefulness is, however, limited by the variety of side effects [283]. Development of more selective molecules, such as GluN2B antagonists, would provide neuroprotective actions with minimal side effects and better tolerability [284,285]. Other approaches would include the discovery of modulators of the glycine site associated with NMDA receptors, of the reuptake systems, as well as of mGlu receptor allosteric modulators to provide fine tuning of the glutamatergic neurotransmission [9,41,110]. Another fundamental issue, in view of the stringent relationship existing between the effects of Glu and the maintenance of the steep extracellular/intracellular concentration gradient, consists in evaluating whether conditions leading to the disruption of the blood brain barrier or the gut blood barrier, such as those induced by dysbiosis or stress, may induce excessive increase of extracellular Glu levels, sustaining development of either CNS and/or ENS disorders, as observed after brain and gut injury [66,99].

An innovative and intriguing approach is represented by the possibility to modulate glutamatergic pathways along the microbiota-gut-brain axis by influencing the microbiota composition. One of the possible approaches in this field is the use of probiotics, which are beneficial bacteria yielding positive health outcomes, and mostly represented by Bifidobacterium and Lactobacillus families [286,287]. In recent years, development of psychobiotics, i.e., neuroactive molecules produced by probiotics [288,289], modulating the levels of circulating tryptophan and the downstream Kyn pathway, has been proposed as a possible approach to for neuroprotection peripherally and in the CNS [38]. This could represent an interesting choice for adjuvant treatment of some IBD and IBS symptoms associated with changes in the levels of Kyn pathway metabolites along the brain-gut axis. In view of the wide range of neuroactive molecules produced by bacteria and the complex interplay existing among them, a promising strategy will be to combine different methodological approaches of metabolomics, metagenomics and metatranscriptomics and proteomics in order to identify bacteria and bacterial genes involved in the modulation of Glu signaling and to verify their potential efficacy as adjuvant in the therapy of gut-brain axis related disorders.

## 8. Conclusions

The ability of the enteric commensal flora to adapt to changes in the host life-style (caused by diet, drugs, social, ethnic and environmental factors) is elevated and strengthen the concept that our behavior may deeply influence this symbiotic organ. There is now strong evidence that the host may communicate with the microbiota by releasing neuroactive molecules, which are recognized by commensal bacteria. Conversely, microbes inhabiting our body may interfere with gut and brain functions by releasing bioactive molecules, via humoral, endocrine, immune and neuronal pathways. In this latter context, it is now widely acknowledged that microbial strains may produce neuroactive molecules such as neurotransmitters (i.e., noradrenaline, dopamine, serotonin, GABA and glutamate) and metabolites, (i.e., tryptophan metabolites) which sustain a possible interkingdom communication system between eukaryota and prokaryota. Glu represents one of the numerous neuroactive molecules active in this interkingdom communication. Indeed, Glu as a neuromodulator/neurotransmitter is involved in the regulation of the microbiota-gut-brain axis. Modulation of glutamatergic neurotransmission influences important physiological functions both in the CNS (learning and memory) and in the ENS (visceral sensitivity and motility). In addition, dysfunction of glutamatergic pathways may represent a critical factor in the pathogenesis and/or in the clinical presentation of a number of CNS disorders, as well as of gastrointestinal diseases, such as IBD and IBS, which display high psychiatric co-morbidity. In this view, studies aiming to clarify the role of Glu along the microbiota-gut-brain axis may eventually lead to the discovery of molecules with a potential therapeutic interest in the treatment of these chronic gastrointestinal pathologies, and the related CNS disorders, such as anxiety and depression. In particular, research in this field opens an exciting scenario on the possibility to target the glutamatergic neurotransmission, by means of traditional pharmacological approaches as well as by the use of neuroactive molecule-producing probiotics as new potential therapeutic tools addressed to the treatment of neurogastrointestinal and/or psychiatric disorders.

## Figures and Tables

**Table 1:** Bacteria producing Glu.

BACTERIA	DESCRIPTION	GLU PRODUCTION	REFERENCES
*Arthrobacter mysorens*	Gram-positive soil bacterium	used for industrial production of l-glutamate	[58]
*Brevibacterium spp.*	Gram-positive soil bacteria	glutamic acid producer	[59,60]
*Corynebacterium glutamicum*	Gram-positive soil bacterium	used industrially for large-scale production of l-glutamic acid	[61]
*Corynebacterium callunae*	Gram-positive soil bacterium	used industrially for large-scale production of l-glutamic acid	[62]
*Lactobacillus plantarum*	Gram-positive bacterium commonly found in many fermented food products, as well as in saliva. The high levels of this organism in food make it an ideal candidate as a probiotic	glutamic acid producer	[53]
*Lactococcus lactis*	Gram-positive used for centuries for fermentation of food. Other than its important function in food, *L. lactis* has become the model LAB when it comes to genetic engineering	glutamic acid producer	[51]
*Methylobacillus sp.*	Group of Gram-negative methylotrophic aerobic bacteria, found in marine and fresh-water ecosystems	glutamic acid producer	[63]

**Table 2:** Nomenclature of Glu transporters.

	Hugo Name	Aliases	REFERENCES
*Plasma membrane transporters*			
	Excitatory amino acid transporter 1 (EAAT1; slc1 a3)	GLAST	[66,70]
	Excitatory amino acid transporter 2 (EAAT2; slc1 a2)	GLT-1	[66,70]
	Excitatory amino acid transporter 3 (EAAT3; slc1 a1)	EAAC1	[66,70]
	Excitatory amino acid transporter 4 (EAAT4; slc1 a6)		[66,70]
	Excitatory amino acid transporter 5 (EAAT5; slc1 a7)		[66,70]
*Vesicular transporters*			
	Vesicular glutamate transporter 1 (VGLUT1; slc17 a7)	______	[68,69]
	Vesicular glutamate transporter 2 (VGLUT2; slc17 a6)	______	[68,69]
	Vesicular glutamate transporter 3 (VGLUT1; slc17 a8)	______	[68,69]
